# Correlation transfer function analysis as a biomarker for Alzheimer brain plasticity using longitudinal resting-state fMRI data

**DOI:** 10.1038/s41598-023-48693-2

**Published:** 2023-12-06

**Authors:** Doaa Mousa, Nourhan Zayed, Inas A. Yassine

**Affiliations:** 1 Computers and Systems Department, Electronics Research Institute, Cairo, Egypt; 2 Mechanical Engineering Department, The British University in Egypt, Cairo, Egypt; 3 Systems and Biomedical Engineering Department, Cairo University, Giza, Egypt

**Keywords:** Neurology, Engineering

## Abstract

Neural plasticity is the ability of the brain to alter itself functionally and structurally as a result of its experience. However, longitudinal changes in functional connectivity of the brain are still unrevealed in Alzheimer’s disease (AD). This study aims to discover the significant connections (SCs) between brain regions for AD stages longitudinally using correlation transfer function (CorrTF) as a new biomarker for the disease progression. The dataset consists of: 29 normal controls (NC), and 23, 24, and 23 for early, late mild cognitive impairments (EMCI, LMCI), and ADs, respectively, along three distant visits. The brain was divided into 116 regions using the automated anatomical labeling atlas, where the intensity time series is calculated, and the CorrTF connections are extracted for each region. Finally, the standard t-test and ANOVA test were employed to investigate the SCs for each subject’s visit. No SCs, along three visits, were found For NC subjects. The most SCs were mainly directed from cerebellum in case of EMCI and LMCI. Furthermore, the hippocampus connectivity increased in LMCI compared to EMCI whereas missed in AD. Additionally, the patterns of longitudinal changes among the different AD stages compared to Pearson Correlation were similar, for SMC, VC, DMN, and Cereb networks, while differed for EAN and SN networks. Our findings define how brain changes over time, which could help detect functional changes linked to each AD stage and better understand the disease behavior.

## Introduction

Alzheimer's disease (AD), a form of neurodegenerative disorders, is a major public health concern due to the growing number of AD patients worldwide^1^. It is also a progressive disease, which worsens with time. It is believed to begin 20 years or more before the appearance of symptoms. According to Centers for Disease Control and Prevention (CDC) reports, the mortality rate due to AD and other dementias in 2020 was approximately 16% more than expected^2^. Typically, AD is identified depending on the medical history of individuals and their families, physical and neurological examinations, blood tests, and brain imaging^2^. Among the brain imaging techniques, resting-state functional MRI (rs-fMRI) has been widely used, in recent studies, to assess AD progression^3^.

Functional MRI measures variations in blood oxygenation level-dependent (BOLD) signals to determine brain activity. It has been suggested that the functional changes likely precede structural alterations^4^. Thus, as revealed by rs-fMRI, low-frequency fluctuation of the BOLD signals provides a valid method for assessing the network functional integrity of structurally segregated brain areas. Different studies have demonstrated rs-fMRI's effectiveness in identifying AD stages vs. NC using cross-sectional datasets^5–9^. However, cross-sectional studies cannot detect the developments or changes in the characteristics of brain activation over time. Therefore, longitudinal studies are essential to understand the dynamic alterations in brain structure and function during AD progression.

Longitudinal studies mainly focuses on investigating the activation changes in specific brain regions related to AD, like the hippocampus and related medial temporal lobe structures, using rs-fMRI with statistical tests like t-test and analysis of variance (ANOVA) test. Yao et al.^10^ studied the role of the amygdala-cortical circuit in the progression of mild cognitive impairment (MCI). They tested the significance of the amygdala using a statistical t-test on 13 MCI subjects along two visits; named baseline and follow-up after nearly 13 months. Their findings indicate that impairments in the functional connectivity of the amygdala may serve as a biomarker for MCI progression. O’Brien et al.^11^ demonstrated that clinical decline in subjects with MCI is related to the loss of hippocampal activation. They applied statistical t-test and ANOVA test on fMRI dataset; formed of 51 older individuals to assess the change in activation between baseline visit and follow-up after 2 years. Bai et al.^12^ examined the changes in default mode network (DMN) function in amnestic MCI (aMCI) and NC subjects, over time, using statistical t-test. Wang et al.^13^ examined the alterations in hippocampal connectivity between 14 MCI and 14 NCs. They revealed that functional connectivity (FC) between the hippocampus and certain regions, including the right frontal lobe, the bilateral temporal lobe, and the right insular, was impeded in MCI. The left posterior cingulate cortex, hippocampus, precuneus, right occipital gyrus, and caudate exhibited increased FC to the hippocampus in MCI. Takao et al.^14^ investigated the long-term (1-year) test–retest reliability of resting-state networks (RSNs) in 31 NC, 63 MCI, and 17 AD through using temporal concatenation group independent component analysis with dual regression. Their findings demonstrate that the test–retest stability of RSNs decays with disease progression, which may reflect the progressive neuro-functional alterations related to the disease pathology. Malotaux et al.^15^ conducted a study to examine the longitudinal alterations in rs-fMRI connectivity among individuals diagnosed with MCI. The participants were categorized based on their amyloid-beta (Aβ) status and clinical progression to dementia over a period of three years. The researchers noted that individuals with progressive MCI had a gradual increase in intra-DMN connectivity with time, as opposed to those with stable MCI. However, no statistically significant alterations were observed in other networks. In addition, the researchers conducted a separate analysis of the anterior and posterior DMN regions. They found that the observed increase in connectivity over time in individuals with progressive MCI was mostly influenced by the front portion of the DMN, whereas the posterior portion did not show a significant increase.

All previously mentioned studies, investigate the role of a certain brain region in the AD progression. So, a longitudinal study that investigates the brain regions during the disease searching for the regions that characterize the disease progression is needed.

This study proposed discovering the brain FC changes in the different AD stages and NC subjects over time. Moreover, we are interested in extracting significant connections that characterize the disease progression in each stage and how they interact together. We hypothesize that, the CorrTF features has the potential to characterize the significant brain regions in the progress of each disease stage. The proposed framework consists of extracting the CorrTF features from the 116 brain regions, among three visits (baseline and two follow-up visits), for each disease stage. Then using a statistical t-test and ANOVA to find the significant brain connections.

## Materials and methods

### Dataset

The dataset was taken from the Alzheimer's Disease Neuroimaging Initiative (ADNI) database. The ADNI was launched in 2003 as a public–private partnership, led by principal investigator Michael W. Weiner, MD. ADNI consisted of participants enrolled at 57 clinical centers in the US and Canada, funded as a private–public partnership. All ADNI studies are conducted according to the Good Clinical Practice guidelines, the Declaration of Helsinki, and U.S. 21 CFR Part 50 (Protection of Human Subjects), and Part 56 (Institutional Review Boards). Written informed consent was obtained from all participants before protocol-specific procedures were performed. The ADNI protocol was approved by the Institutional Review Boards of all of the participating institutions. The main objective of ADNI is to test whether combining different diagnosing techniques can be pooled to characterize the progression of MCI and early AD. For up-to-date information, see www.adni-info.org. A complete description of ADNI is available at http://adni.loni.usc.edu/. Moreover, the data access requests are to be sent to http://adni.loni.usc.edu/data-samples/access-data/.

The rs-fMRI images were collected at baseline, three months, six months, 12 months from baseline, and annually afterward. We did exclude the patients who had not at least two follow-up visits following the baseline scan. In this study, 99 subjects were downloaded from ADNI, formed of four classes: NCs, EMCI, LMCI, and AD patients, as reported in Table 1. The rs-fMRI images were acquired using 3.0 Tesla Philips Achieva scanners. The scanning protocol parameters are reported in Table 2. According to ADNI2 inclusion criteria https://adni.loni.usc.edu/wp-content/uploads/2008/07/adni2-procedures-manual.pdf, the Mini-Mental State Exam (MMSE) score for CN, EMCI, and LMCI is between 24 and 30. While the exam score for AD is between 20 and 26. For all groups, exceptions may be made for subjects with less than 8 years of education at the discretion of the project director.

**Table 1:** Overview of rs-fMRI study groups (mean ± standard deviation).

Study group	No. of subjects	Age range (years)	Sex (male/female)	Visit 1 duration from baseline (months)	Visit 2 duration from baseline (months)
NC	29	74.31 ± 5.83	17/12	8.31 ± 3.37	18.69 ± 6.45
EMCI	23	69.83 ± 6.29	9/14	8.65 ± 3.54	17.26 ± 6.30
LMCI	24	72.92 ± 6.70	15/9	3.04 ± 0.36	6.46 ± 0.51
AD	23	74.70 ± 6.20	12/11	3.22 ± 0.52	6.48 ± 0.79

**Table 2:** rs-fMRI scanning protocol parameters.

Parameter	Value
Echo time (TE)	30 ms
Repetition time (TR)	3000 ms
Flip angle	80°
Pixel size	3.3 × 3.3 mm
Acquisition matrix size	64 × 64
Slice thickness	3.3 mm
Number of slices/volumes	48/140

## Methodology

### Data preprocessing and brain network analysis

**Figure 1:**
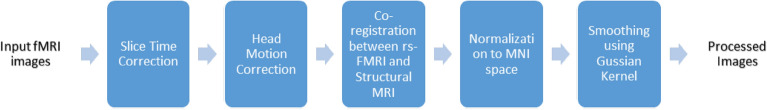
Summarization of the pre-processing steps.

The typical preprocessing procedures for rs-fMRI are carried out, as shown in Fig. 1, using the software tool Statistical Parametric Mapping SPM12 (Welcome Trust Centre for Neuroimaging, London, UK)^16^. It involves discarding the first ten time-point volumes for each subject in order to ensure magnetization equilibrium. The remaining volumes are then corrected for the interleaved order of slices, in which the all odd-number slices were collected first and then all even-numbered slices. Registration for head motion artifact elimination, and co-registration of functional and structural images has been later applied. The images are then normalized to standard space using the SPM12 MNI/EPI (Montreal Neurological Institute/Echo Planer Image) template. The images were spatially smoothed using a 5 mm FWHM Gaussian kernel to increase the signal to noise ratio. Each volume was then segmented into 116 regions of interest (ROIs), as found in^9^, according to automatic anatomical atlas labeling (AAL)^17–19^. The different ROI's mean intensity time series was then obtained and band-pass-filtered at 0.01–0.08 Hz to better localize the rs-fMRI while removing the noise signals as a result of some psychological artifacts such as breathing, and heartbeat. Finally, each subject was expressed by a matrix with 116 (number of regions) × 130 (time points), defining the time signal for each ROI.

### CorrTF feature extraction

The correlation transfer function (CorrTF) quantifies the amount of information transmitted between the input and output ROIs. Therefore, the properties of the functional connection path between any two regions can be anticipated. AD is characterized by nerve cell death, affecting the region's connection path. As a result, alterations in the connection path may potentially differentiate between healthy and diseased cases^9,20,21^ and provide critical information about changes in brain connectivity over time. CorrTF feature extraction technique can be considered as a biomarker for AD stage identification^9^.

Theoretically, the transfer function models the system’s output for each possible input^9^. The relationship between output *y(t)* and input *x(t),* for any system, can be modeled using

1 $$y(t)=\underset{-\infty }{\overset{\infty }{\int }}x\left(\tau \right)h(t-\tau )d\tau$$

where the *h(t)* is the impulse response that defines the system behavior. While the relationship in the frequency domain can be modeled using

2 $$Y\left(f\right)=X\left(f\right)H(f)$$

where *Y(f)*, *X(f)* and *H(f)* are the Fourier transform of the *y(t), x(t),* and *h(t)* respectively. Similarly, this definition can be interpreted to model the connectivity path between any pair of brain regions, as illustrated in

3 $$CorrTF\left({ROI}_{1},{ROI}_{2}\right)=\left|\frac{\mathcal{F}({ROI}_{1})}{\mathcal{F}({ROI}_{2})}\right|$$

where *CorrTF (ROI*_*1*_*, ROI*_*2*_*)* is the transfer function calculated between the mean time series of *ROI*_*1*_ and *ROI*_*2*_,* Ƒ* is the discrete Fourier Transform.

### Statistical analysis

A standard t-test and ANOVA were employed to explore the between-group changes in CorrTF connections activation over time at a significance level of p < 0.05. The data were normalized, so that the sum of the squares equal one, to fulfill the normal distribution condition for the t-test. For NC and each disease stage, the statistical significance of every CorrTF connection, along three visits, was examined. The connection is considered significant if there were significant differences in activation between both pairs; baseline-visit1 and visit1-visit2.

## Results

In this paper, we are interested in extracting the significant connections that characterize the progression of AD in each stage. A standard t-test and ANOVA test were employed to explore the between-group changes in CorrTF connections activation over time, at a significance level of p < 0.05. Almost all connections extracted using t-test included within connections extracted using ANOVA test. Consequently, we will discuss the common connections extracted using both tests. The connections regions names and their abbreviations have been listed in Table 3.

**Table 3:** List of the 116 brain regions and their abbreviations.

ROI label	Abb	ROI label	Abb	ROI label	Abb
Amygdala	AMYG	Frontal_Mid_Orb	ORBmid	Precuneus	PCUN
Angular	ANG	Frontal_Mid	MFG	Putamen	PUT
Calcarine	CAL	Frontal_Sup_Medial	SFGmed	Rectus	REC
Caudate	CAU	Frontal_Sup_Orb	ORBsup	Rolandic_Oper	ROL
Cerebelum_10	CRBL10	Frontal_Sup	SFGdor	Supp_Motor_Area	SMA
Cerebelum_3	CRBL3	Fusiform	FFG	SupraMarginal	SMG
Cerebelum_4_5	CRBL45	Heschl	HES	Temporal_Inf	ITG
Cerebelum_6	CRBL6	Hippocampus	HIP	Temporal_Mid	MTG
Cerebelum_7b	CRBL7b	Insula	INS	Temporal_Pole_Mid	TPOmid
Cerebelum_8	CRBL8	Lingual	LING	Temporal_Pole_Sup	TPOsup
Cerebelum_9	CRBL9	Occipital_Inf	IOG	Temporal_Sup	STG
Cerebelum_Crus1	CRBLCrus1	Occipital_Mid	MOG	Thalamus	THA
Cerebelum_Crus2	CRBLCrus2	Occipital_Sup	SOG	Vermis_10	Vermis10
Cingulum_Ant	ACG	Olfactory	OLF	Vermis_1_2	Vermis12
Cingulum_Mid	DCG	Pallidum	PAL	Vermis_3	Vermis3
Cingulum_Post	PCG	ParaHippocampal	PHG	Vermis_4_5	Vermis45
Cuneus	CUN	Paracentral_Lobule	PCL	Vermis_6	Vermis6
Frontal_Inf_Oper	IFGoperc	Parietal_Inf	IPL	Vermis_7	Vermis7
Frontal_Inf_Orb	ORBinf	Parietal_Sup	SPG	Vermis_8	Vermis8
Frontal_Inf_Tri	IFGtriang	Postcentral	PoCG	Vermis_9	Vermis9
Frontal_Med_Orb	ORBsupmed	Precentral	PreCG		

**Figure 2:**
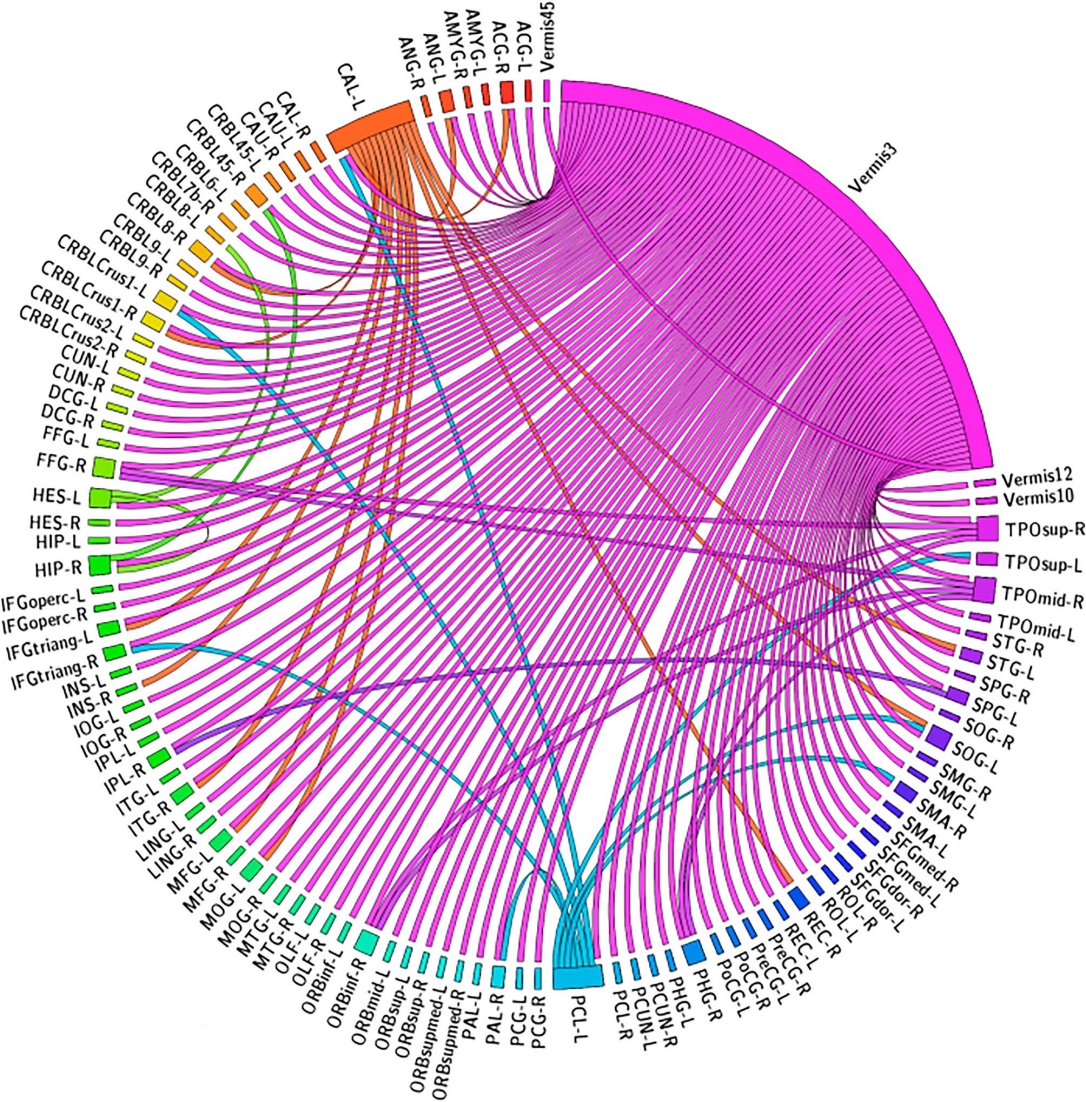
The significant CorrTF connections that characterize the EMCI progression. Connection were colored based on the originated ROI color. The figure has been generated using the CIRCOS online tool (http://mkweb.bcgsc.ca/tableviewer/).

Figure 2 shows the 133 significant CorrTF connections that characterize the EMCI progression. Among these connections, we found that the connections were mainly directed only from eight brain regions which are; Vermis_3, Left CAL, Left PCL, Right TPOmid, Right TPOsup, Left HES, Right HIP, and Left SPG. The number of connections directed from each region are: 101, 12, 10, 3, 3, 2, 1, and 1 respectively.

**Figure 3:**
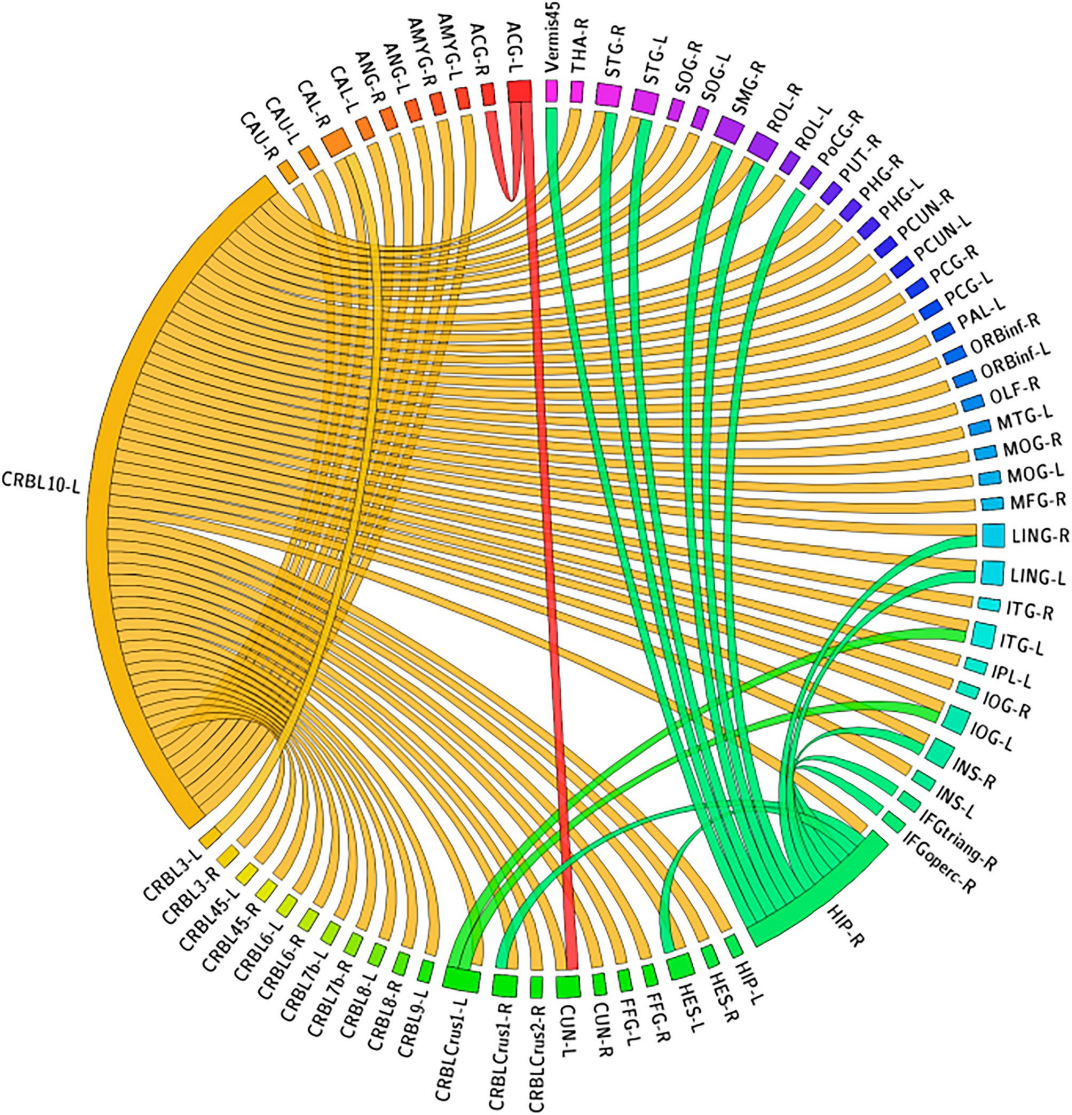
The significant CorrTF connections that characterize the LMCI progression. Connection were colored based on the originated ROI color. The figure has been generated using the CIRCOS online tool (http://mkweb.bcgsc.ca/tableviewer/).

Figure 3 shows the 79 significant CorrTF connections that characterize the LMCI progression. Among these connections, we found that the connections were mainly directed only from five brain regions which are; Left CRBL10, Right HIP, Left CRBLCrus1, Left ACG, and Left CRBL3. The number of connections directed from each region are: 61, 13, 2, 2, and 1 respectively.

**Figure 4:**
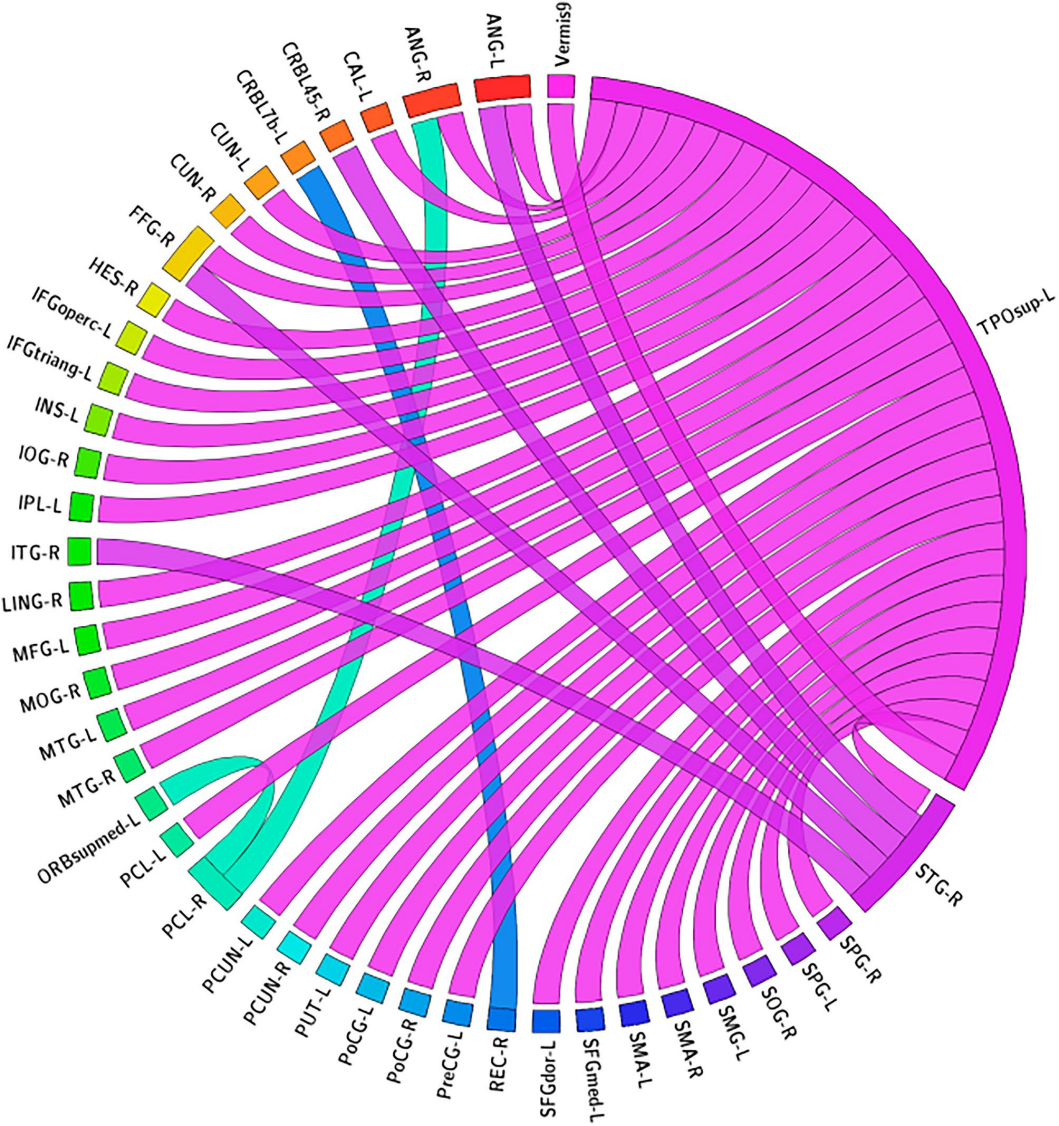
The significant CorrTF connections that characterize the AD progression. Connection were colored based on the originated ROI color. The figure has been generated using the CIRCOS online tool (http://mkweb.bcgsc.ca/tableviewer/).

Figure 4 presents the 41 significant CorrTF connections that characterize the AD progression. Among these connections, we found that the connections were mainly directed only from four brain regions: left TPOsup, Right STG, Right PCL, and Right REC. The number of connections directed from each region are: 34, 4, 2, and 1 respectively. At all connections, the t-test critical value sign changed from positive to negative for the difference between baseline-visit1 and visit1-visit2, respectively, except the connections directed from Right STG changed in the opposite direction. In contrast to the previously mentioned stages of AD, the NC showed no significant statistical difference in any CorrTF connections between baseline-visit1 and between visit1-visit2.

**Figure 5:**
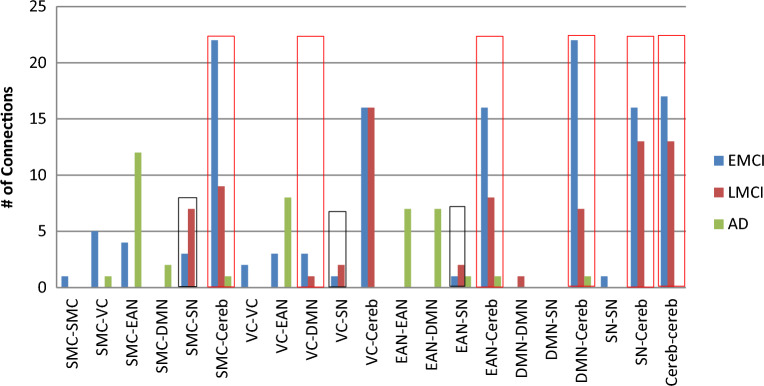
Comparison between the numbers of significant connections, grouped by input–output networks with connection’s directionality ignored, for different AD stages. *SMC* sensorimotor cortex, *EAN* executive attention network, *VC* visual cortex, *Cereb* cerebellum, *DMN* DefaultMode network, *SN* subcortical nuclei.

In Fig. 5, we grouped the significant connections to highlight the most altered network pairs among all AD stages. As observed from Fig. 5., the Cerebellum network has the highest contribution with 178 connections. It is worth noting that Figs. 2, 3, and 4 were generated using the CIRCOS tool^22^.

**Figure 6:**
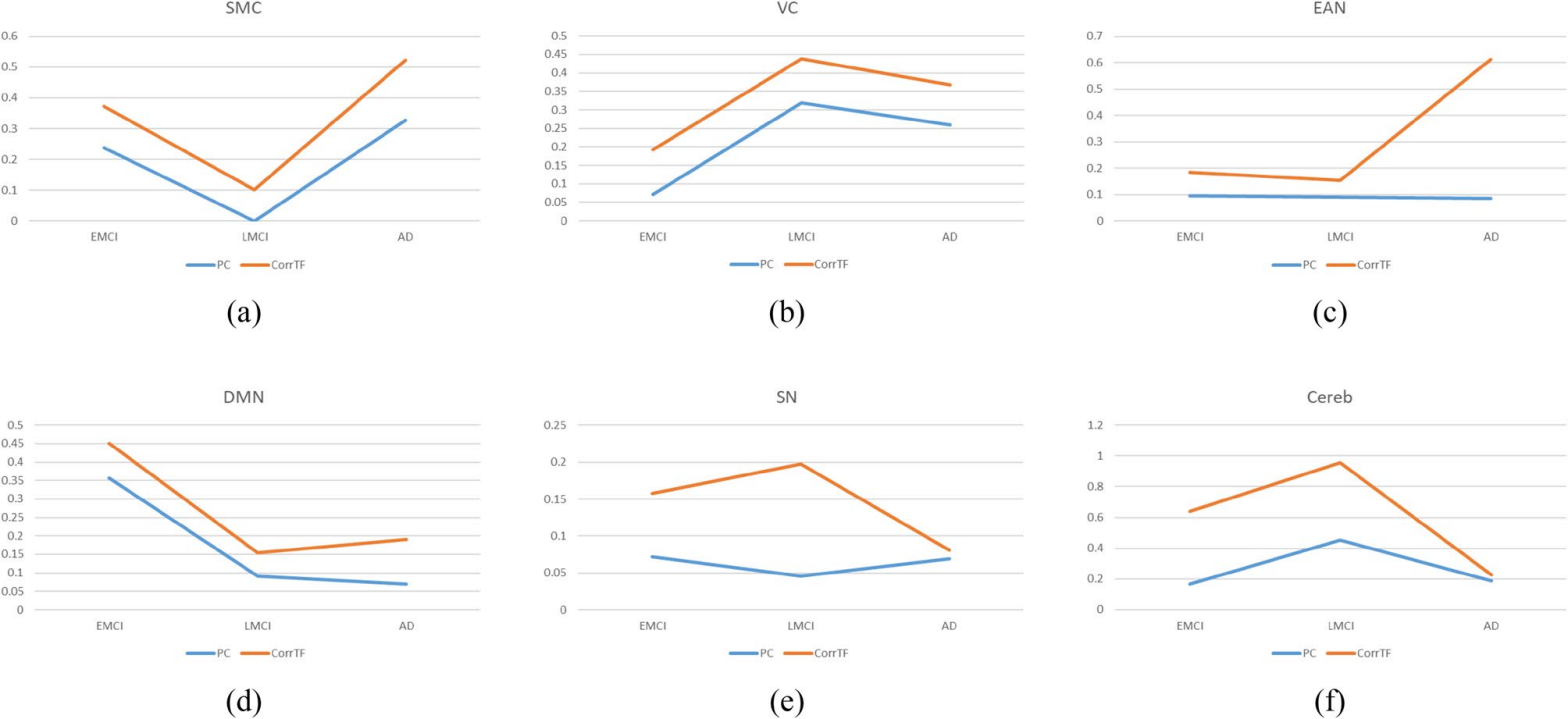
Comparison between percentage of significant contribution for each functional network using CorrTF (red) and Pearson Correlation (blue) for each disease stage: (**a**) SMC, (**b**) VC, (**c**) EAN, (**d**) DMN, (**e**) SN, and (**f**) Cereb.

A comparison between the proposed method using CorrTF and Pearson correlation (PC)^23^ is found in Fig. 6. The comparison was done to validate our results using CorrTF vs PC, the conventional method used in the literature. To increase the normality of the correlation coefficients, we first computed the PC for interregional connectivity. Fisher's z-transformation was then applied to standardize the PC feature values. Figure 6 presents the percentage of contribution for each functional network acquired using PC vs. CorrTF for each disease stage. The patterns of longitudinal changes among disease stages for both techniques were similar, for SMC, VC, DMN, and Cereb networks, while differed for EAN and SN networks.

## Discussion

This longitudinal fMRI study demonstrates the significant CorrTF connections that characterize the progression of each AD stage. It is worth noting that, while MCI is a risk factor for AD it is not a guarantee of developing the disease. In fact, most people with MCI will not go on to develop AD^24^. There are a number of things that people with MCI can do to reduce their risk of developing AD, such as staying mentally and physically active, eating a healthy diet, and getting enough sleep. In our manuscript, the progression of AD stages is represented by tracking the longitudinal changes in cognitive cohorts occurred such as those with probable AD dementia (established clinically rather than biologically) and a potential prodromal cohort (E/L MCI).

### Analysis of EMCI progression

Figure 2 shows the significant connections that characterize the EMCI progression. Among these connections, there are 101 connections between different brain regions and Vermis_3. The Vermis is a part of the cerebellum involved in motor control, cognition, and emotional regulation^25^. Several studies suggest the relation between cerebellum Vermis and cognitive impairment and other symptoms of neurodegenerative diseases^26–28^. As Cerebellum Vermis may be involved in the visuospatial functions^26^, which may be impaired at the early stages of AD^29^. Consequently, we interpret that the significant contribution of vermis may be a compensatory activation related to the cognitive changes occurred to the EMCI subjects. Besides the Vermis, areas of SMC, such as the Left Paracentral Lobule, Left Superior Parietal Gyrus, Left Heschl Gyrus, and Right Superior Temporal Pole, are significantly contributed by 16 connections. Several studies investigated the role of SMC in the early AD stages^30–34^. Moreover, the connections within these particular regions are consistent with the literature^35–42^. Gupta et al.^43^ demonstrate that the increased significance in Paracentral Lobule could be attributed to maintaining motor function performance. Additionally, Fig. 2 shows the contribution of Calcarine, a component of the visual cortices. Visual disturbance, like spatial contrast sensitivity, impairment in visual acuity, color sensitivity, and blurred vision, is expected in AD patients^44^. Li et al.^45^ found a high activation in Left Calcarine Cortex for MCI compared to AD. This could be a self-adaptive or decompensating mechanism, which agrees with our findings. Furthermore, four significant connections are directed from two SN regions; Right Hippocampus and Right Middle Temporal Pole. Various studies confirmed our results, that there are significant changes in the volume of the Middle Temporal Pole and Hippocampal regions^46–48^. Tang et al.^49^ considered changes in the structure of the hippocampus and subcortical nuclei as a sign of the upcoming transformation of MCI. O’Brien et al.^11^ demonstrated that the increased Hippocampal FC implied compensatory mechanisms for memory loss.

### Analysis of LMCI progression

Figure 3 presents the significant connections that characterize the LMCI progression. The most significant connections were directed from areas of the cerebellum; Left Cerebellum_10, Left Cerebellum_3 and Left Cerebellum Crus_1. This is consistent with the symptoms of decline in motor, cognition, and emotional activities since the cerebellum regulate these functions^50^. Recently, researchers have given attention to the alterations of cerebellum regions during AD’s different stages^51^. Tang et al.^52^ indicate significant changes regarding the FC of cerebellar cognitive subregions within the AD and MCI groups. The strength of left cerebellar FC is positively associated with certain cognitive subsites; memory, executive function, visuospatial function, and global cognition in AD and aMCI^53^. Hoxha et al.^54^ concluded that the Cerebellum region is vulnerable to amyloid-β toxic destruction, even at the onset of the disease, resulting in impaired motor function. Jacobs et al.^55^ state that the cerebellum is more than a silent witness in the pathophysiology of AD and its clinical phenomenology. Besides the cerebellum, there are 13 connections directed from Right Hippocampus. Compared to EMCI, the HIP connections participate more in the LMCI progression. This may be considered a compensatory action from the brain to restore the loss of memory function, as suggested by Ref.^11^. Figure 3, also, shows the significant connections directed from Left Anterior Cingulate Gyrus. The Cingulate Gyrus is an essential part of the limbic system involved in regulating cognitive function^56^. Various studies investigate the relation between cognition impairment and Cingulate Cortex^56–59^. Wei et al.^60^ found that the progressive MCI group had smaller left posterior and caudal Anterior Cingulate than the stable MCI group at baseline. The Anterior Cingulate Cortex is involved in central cognitive functions, such as motivation, decision-making, education, cost–benefit analysis, and conflict and problem solving^56^, which match the disease symptoms.

### Analysis of AD progression

Figure 4 shows the significant CorrTF connections that identify the AD progression. The most significant connections were directed from the Left Superior Temporal Pole (TPOsup), a region of the EAN. The EAN is required for active maintenance of and manipulation of information in working memory as well as for principle problem solving and decision making^61^. Authors in^61^ found that the FC within the EAN increased a bit in the MCI patient while declined significantly in the AD patients. Chen et al.^62^ found that AD patients suffer from abnormal left TPOsup. Besides left TPOsup, Right Superior Temporal Gyrus, and Right Paracentral Lobule, areas of SMC are also associated with AD, as shown in figure. Zhan et al.^63^ indicated abnormal network components in DMN, SMC, visual-sensory network, and visual-attention network during AD progression. Different researches demonstrate the abnormality of STG in AD patients^64–67^. Xiao et al.^64^ reported that during a span of one year, individuals diagnosed with AD had notable degeneration in both STG and the left caudate. Furthermore, a reduction in grey matter volume in the right STG and left caudate was found to be associated with a drop in cognitive function. Clarke et al.^68^ concluded that the high-risk AD group was related to a hub in the right paracentral lobe, a medial frontoparietal cortical area with sensorimotor functions. Figure 4 also shows a significant connection directed from the Right Rectus Gyrus, the area of DMN. Li et al.^69^ stated that MCI patients exhibited considerably lower clustering coefficients in the right inferior parietal gyrus, right superior parietal gyrus, right rectus and, left middle frontal gyrus as well as lower shortest path length in the left paracentral lobule, compared to NC. Yang et al.^70^ demonstrated that following donepezil treatment, patients with AD exhibited increased amplitude of low-frequency fluctuations, measured using rs-fMRI, in the right gyrus rectus, which decreased after treatment.

Among the significant connections in our findings, the t-test critical value changed from positive to negative except for the connection to Right STG in AD progression. Thus, we can interpret that this connection gets worth after specific compensation, whereas the other connections compensate for function loss as time progresses. In this context and according to the findings of Xiao et al.^64^, people diagnosed with Alzheimer's disease (AD) saw significant degradation in both the STG and the left caudate over the course of one year. Moreover, a decrease in grey matter volume in the right STG and left caudate nucleus was observed to be correlated with a decline in cognitive performance.

### Analysis across different AD stages

The number of significant connections extracted for EMCI, LMCI, and AD was 133, 79, and 41, respectively, as observed in Figs. 2, 3, and 4. This observation interprets that the number of connections employed by the brain to transfer information is inversely proportional to the disease severity. With the disease progression, the brain FC may lose some of its compensation ability due to pathological changes. This may relate to the disease’s progressive nature, which means its symptoms become worse with time.

Figure 5 presents the number of significant connections between each pair of networks for all AD stages with directionality ignored. We observed that the significant connections decrease with disease severity in six groups: SMC-Cereb, VC-DMN, EAN-Cereb, DMN-Cereb, SN-Cereb, and Cereb–Cereb, highlighted by red rectangles. These results define the clinical relationship between motor and cognitive function deterioration in AD progression^71–73^. Zheng et al.^73^ reported that The Crus II of the cerebellum was functionally connected to several DMN regions and frontoparietal network (FPN) regions. It was also reported that the lobule IX of the cerebellum was involved in the DMN, FPN, VC, and SMC regions. While Halko et al.^72^ reveal that altering activity in the lateral cerebellar Crus I/II impacts the cerebral DMN, however vermal lobule VII stimulation affects the cerebral dorsal attention system. We can also observe that the Cerebellum regions are highly affected by AD. Hoxha et al.^54^ stated that the cerebellum is considered a region exposed to amyloid-β toxic destruction, even at the onset of the disease, with motor function implications.

In contrast, we observed, from Fig. 5, that the number of significant connections increased in LMCI from EMCI in the relation between the SN network and three other networks: SMC, VC, and EAN, highlighted by black rectangles. Generally, We observed an increase in the hippocampus, area of SN network, connectivity in LMCI compared to EMCI that was missed in the final AD stage. This may be considered a compensation action during the AD progression, which failed at the AD final stage, similar conclusion suggested by authors in^13^.

Figure 6 shows the percentage of significant contribution by each functional network obtained using Pearson Correlation vs. CorrTF. The percentage of contribution is the ratio of contributed regions to the total significant regions. For both techniques, the pattern of longitudinal changes among disease progression was quite similar in SMC, VC, DMN, and Cereb networks. However, the longitudinal pattern of change differed for EAN and SN networks. This comparison supports our interpretations and understanding of the longitudinal changes during the disease progression.

### Limitations

There are several limitations in our study. First the dataset doesn’t include subjects who known to be progressed to next severe stage e.g. subjects progressed from EMCI to LMCI or from LMCI to AD. Second, the proposed work investigates only the significance of one factor which is the CorrTF features. It will be very valuable to include other factors like age, and sex as covariates in the analysis. Finally, the study's sample size is comparatively limited and the age range of the four groups is of high variance. These perhaps impacting the findings to a certain degree. In future work, large number of samples with appropriate age range will be proposed.

Although the labeled downloaded ADNI date was enough to our study goal to investigate the longitudinal changes in cognitive cohorts one of our future work goals will be checking fluidic/PET biomarkers or to make sure that the categorization was done according to the biological classes.

## Conclusion

This study investigates the significant connections that characterize each AD stage using the CorrTF features extracted from rs-fMRI data. Two statistical tests were employed to find the significant connections with time among three different visits to the hospital. For NC subjects, we find no significant connections along three visits. The most significant connections were mainly directed from cerebellum regions for EMCI and LMCI. While in AD, the cerebellum region has no significant connections. Our results suggest that the Cerebellum regions are highly affected by AD progression as their connectivity decreases while the disease worsens. Additionally, the hippocampus showed certain compensation ability in the early stages while it failed in the late stage. Further investigations to CorrTF connections to highlight the contribution of some regions rather than others in the disease and during progression may also be considered in both Alzheimer’s disease and other neurological disorders.

## Data Availability

The datasets analyzed during the current study are owned by a third-party organization “Alzheimer’s disease Neuroimaging Initiative” (ADNI) database. Researchers can request and access the data through the website of the Alzheimer’s Disease Neuroimaging Initiative (ADNI) (http://adni.loni.usc.edu). Authors had no special access privileges to this data.
